# Epidemiology and molecular typing of multidrug-resistant bacteria in tertiary hospitals and nursing homes in Flanders, Belgium

**DOI:** 10.1007/s10096-023-04699-2

**Published:** 2023-11-16

**Authors:** Stefanie van Kleef-van Koeveringe, Veerle Matheeussen, Annette Schuermans, Sien De Koster, Natascha Perales Selva, Hilde Jansens, Dieter De Coninck, Katrien De Bruyne, Klaas Mensaert, Marjolein Kluytmans-van den Bergh, Jan Kluytmans, Herman Goossens, Wouter Dhaeze, Isabel Leroux-Roels, on behalf of the i-4-1-Health Study Group

**Affiliations:** 1 Laboratory of Medical Microbiology, University Hospital Antwerp, Edegem, Belgium; 2 Department of Medical Microbiology, Vaccine & Infectious Disease Institute (VAXINFECTIO), University of Antwerp, Wilrijk, Belgium; 3 Department of Infection Control and Epidemiology, University Hospital Leuven, Leuven, Belgium; 4 Department of Infection Control, University Hospital Antwerp, Edegem, Belgium; 5 bioMérieux, Augmented Diagnostics, Industrial Microbiology, Applied Maths NV, Sint-Martens-Latem, Belgium; 6 Department of Infection Control, Amphia Hospital, Breda, The Netherlands; 7 Julius Center for Health Sciences and Primary Care, UMC Utrecht, Utrecht, The Netherlands; 8 Microvida Laboratory for Microbiology, Amphia Hospital, Breda, and Department of Medical Microbiology, University Medical Center Utrecht, Utrecht, The Netherlands; 9 Department prevention, Agentschap Zorg en Gezondheid, Leuven, Belgium; 10 Laboratory of Medical Microbiology, University Hospital Ghent, Ghent, Belgium

**Keywords:** MDRO, Screening, Whole-genome sequencing, wgMLST, Molecular typing, Transmission

## Abstract

This study aimed to map MDRO carriage and potential transmission within and between three Flemish tertiary care hospitals and their neighbouring nursing homes. A cross-sectional MDRO prevalence survey was organized between October 2017 and February 2019. Perianal swabs were cultured for detection of MDRO. Determination of clonal relatedness based on wgMLST allelic profiles was performed. The prevalence of MDRO in Belgian hospitals and NHs is on the rise, compared to previous studies, and transmission in and between institutions is observed. These results re-emphasize the need for a healthcare network-wide infection prevention strategy in which WGS of MDRO strains can be supportive.

**Supplementary Information:**

The online version contains supplementary material available at 10.1007/s10096-023-04699-2.

## Introduction

Increased antimicrobial resistance (AMR) constitutes a major public health concern in healthcare and community settings [1]. At the healthcare facility level, multidrug-resistant organisms (MDRO) can spread by physical contact between patients and healthcare workers or through contaminated surfaces and medical devices. Furthermore, the transfer of patients between hospitals and other healthcare institutions, such as nursing homes (NHs), within and between healthcare networks plays an important role in the spread of MDROs [2]. As data on MDRO carriage in Belgian healthcare institutions are scarce and transmission between institutions has not been studied on a large scale, our study explored ESBL-E, CPE and VRE prevalence and transmission in three tertiary care hospitals and 13 NHs in Flanders, Belgium, using a combination of conventional culture techniques and WGS.

## Materials and methods

### Study design

A cross-sectional MDRO prevalence survey, the i-4-1-Health project, was organized in the cross-border region of the southern part of the Netherlands and Flanders, Belgium. In this paper, the results obtained between October 2017 and February 2019 from the Flemish hospitals (*n* = 3) and NHs (*n* = 13) are presented.

### Sampling and microbiological analysis

The microbiological method of the project is described by Kluytmans – van den Bergh et al. [3].

### Whole-genome sequencing

Sequencing was performed on an Illumina MiSeq sequencer, using the MiSeq reagent kit v2 generating 250-bp paired-end reads. wgMLST was performed using the BioNumerics software v7.6.3. (bioMérieux, France). Clonal relatedness between isolates was determined based on the similarity of wgMLST allelic profiles. By combining both sequencing and epidemiological data of well-described outbreaks, similarity thresholds for clonal relatedness were determined. Sequencing data should be combined with the metadata to make conclusions for an outbreak, especially in the ‘grey zone’(Table S1). Using a BLAST-based approach requiring at least 95% identity with the reference sequence and at least 95% reference length coverage, genes known to confer resistance were identified from the assembled genomes. Reference sequences and mutations from the Center for Genomic Epidemiology’s ResFinder (database version 2019-08-21) and PointFinder (database version 2019-07-02) databases were used, respectively.

## Results

### Sample and patient information

In total, 2536 perianal or stoma swabs were obtained from patients in Flemish tertiary care hospitals (*n* = 1878) and NH residents (*n* = 658). After the exclusion of six swabs (0.2%) due to poor sampling quality and 125 swabs (6.7%) due to duplicate sampling from previously sampled hospitalized patients, 2405 swabs remained for analysis. Of those, 1748 (73%) were obtained in hospitals and 657 (27%) in NHs (Table 1).

**Table 1:** MDRO prevalence in perianal swabs from hospitalized patients (H) and nursing home residents (NH)

	% screened patients or residents	*N* samples	ESBL-E, *n* (%)	CPE, *n* (%)	VRE, *n* (%)
H 1	34.6	485	77 (15.9)	11 (2.3)	3 (0.6)
H 2	79.9	602	69 (11.5)	5 (0.8)	5 (0.8)
H 3	86.0	661	53 (8.0)	2 (0.3)	8 (1.2)
**Total H**	**59.8**	**1748**	**199 (11.4)**	**18 (1.0)**	**16 (0.9)**
NH 1	78.8	82	5 (6.1)	0 (0.0)	0 (0.0)
NH 2	70.4	76	10 (13.2)	0 (0.0)	0 (0.0)
NH 3	60.2	53	6 (11.3)	0 (0.0)	0 (0.0)
NH 4	23.6	17	2 (11.8)	0 (0.0)	1 (5.9)
NH 5	43.2	51	8 (15.7)	0 (0.0)	0 (0.0)
NH 6	56.2	73	8 (11.0)	0 (0.0)	0 (0.0)
NH 7	57.5	65	23 (35.4)	0 (0.0)	0 (0.0)
NH 8	65.5	78	10 (12.8)	0 (0.0)	0 (0.0)
NH 9	55.2	32	1 (3.1)	0 (0.0)	1 (3.1)
NH 10	17.2	15	6 (40.0)	0 (0.0)	0 (0.0)
NH 11	28.8	23	1 (4.3)	0 (0.0)	0 (0.0)
NH 12	61.4	62	6 (9.7)	0 (0.0)	0 (0.0)
NH 13	31.6	30	6 (20.0)	0 (0.0)	0 (0.0)
**Total NH**	**51.6**	**657**	**92 (14.0)**	**0 (0.0)**	**2 (0.3)**
**Total**	**57.3**	**2405**	**291 (12.1)**	**18 (0.7)**	**18 (0.7)**

*H* hospital, *NH* nursing home, *ESBL-E* extended-spectrum β-lactamase-producing *Enterobacterales*, *CPE* carbapemase-producing *Enterobacterales*, *VRE* vancomycin-resistant *Enterococci*

### Prevalence of ESBL-E, CPE and VRE in hospitals and NHs

The percentage of screened patients or residents is based on the number of patients of residents present on a ward. The genotypic confirmed ESBL-E, CPE and VRE isolates are shown in Table 1. Of the MRDOs, ESBL-E were cultured most often (11.4% in hospitals and 14.0% in NHs) compared to a lower prevalence of VRE (0.9% in hospitals and 0.3% in NHs) and CPE (1.0% in hospitals and undetected in NHs). Significant differences in prevalence of MDRO between the individual hospitals (*p* < 0.001) and between NHs (*p* < 0.001) were detected, while no significant variation in prevalence between the hospital and NH sector was observed.

### Phenotype-genotype correlation for MDRO detection

In all VRE (15 *E. faecium* and 3 *E. avium* strains with an MIC for vancomycin of > 4 mg/l), a resistance gene could be detected (14 vanA genes and 4 vanB genes). However, for ESBL-E and CPE, phenotypic resistance could not always be confirmed by the presence of a resistance gene or mutation, resulting in lower genetic confirmation rates, especially for CPE (Table 2). The presence of other β-lactamase genes and porins are usually responsible for an ESBL-like phenotype.

**Table 2:** Confirmation rates of phenotypical ESBL-E and CPE strains by the presence of resistance genes

	ESBL -E			CPE		
	P (*n*)	G (*n*)	*C* (%)	P (*n*)	G (*n*)	*C* (%)
*Escherichia coli*	249	233	93.6	11	3	27.3
*Klebsiella pneumoniae*	69	66	95.7	15	9	60.0
*Enterobacter cloacae* complex	19	17	89.5	6	1	16.7
*Citrobacter* spp.	14	5	35.7	7	7	100
*Proteus* spp.	10	1	10.0	2	0	0.0
*Klebsiella* spp.	7	3	42.9	2	0	0.0
*Klebsiella aerogenes*	6	3	50.0	3	2	66.7
*Hafnia alvei*	2	0	0.0	0	0	-
*Morganella morganii*	0	0	-	2	1	50.0
*Serratia marcescens*	0	0	-	1	0	0.0
**Total**	**376**	**328**	**87.2**	**49**	**23**	**46.9**

*ESBL-E* extended-spectrum β-lactamase-producing *Enterobacterales*, *CPE* carbapenemase-producing *Enterobacterales, P* phenotypically confirmed, *G* genotypically confirmed, *C* genotypic confirmation rate

ESBL-E were detected in 291 of the 2405 swabs, corresponding with 328 ESBL-E strains (predominantly *E. coli* and *K. pneumoniae*) as 37 swabs contained multiple ESBL-E strains. Over half (51.5%) of the ESBL production capability could be attributed to the presence of the *bla*_CTX-M-15_ gene.

**Fig. 1:**
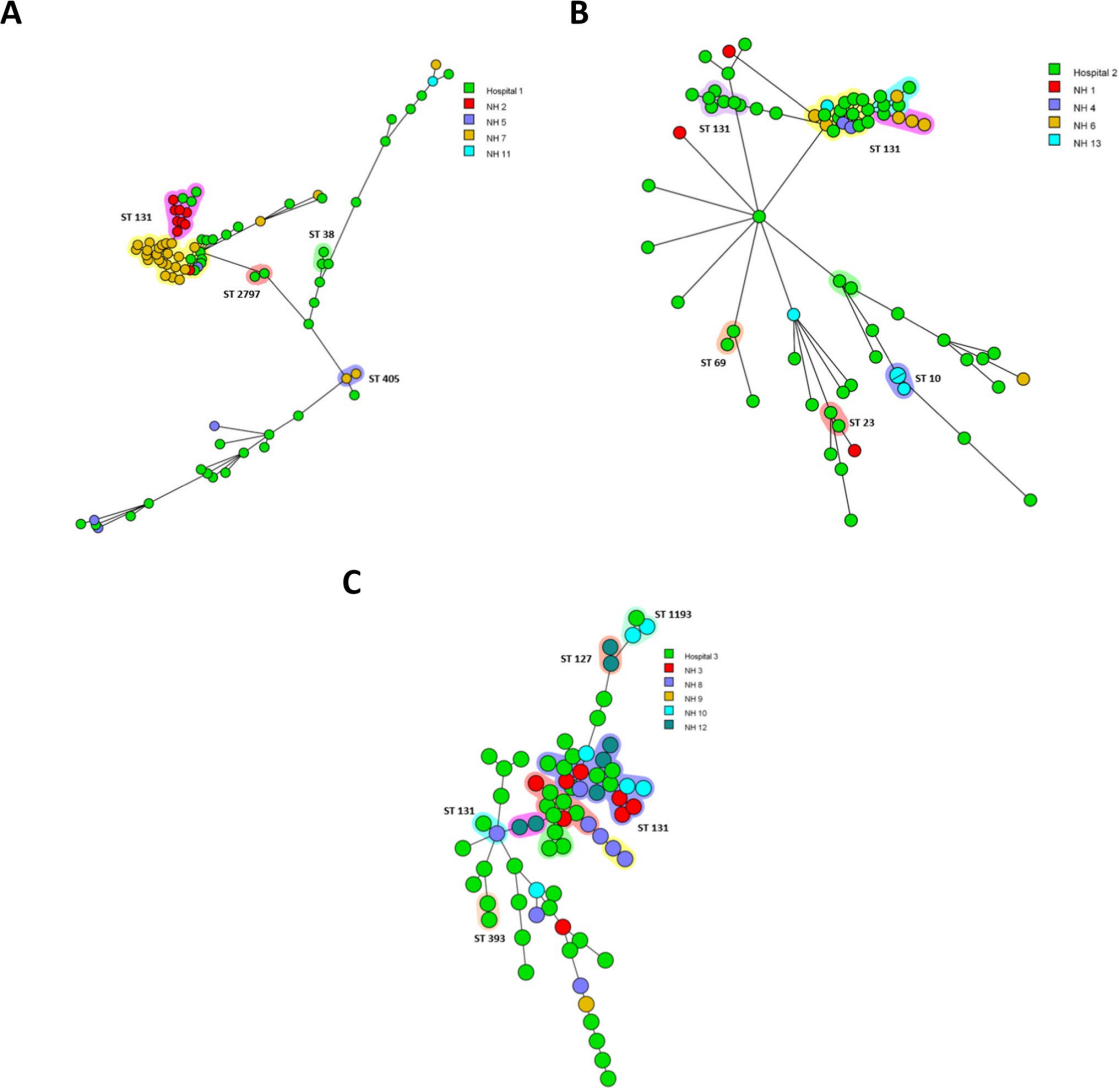
Three minimum spanning trees based on wgMLST analysis of 233 ESBL-*E. coli* isolates per geographical region around the three hospitals (hospital 1 (**A**), hospital 2 (**B**) and hospital 3 (**C**) and their surrounding NHs). Isolates are represented by circles connected by branches proportional to the allelic distance, colours of the circle represent the origin location, the shading represent clonal clusters and ST number represent sequence types

To study local and regional clonal relatedness of ESBL-*E. coli*, separate spanning trees were created for each hospital and its surrounding NHs (Fig. 1A–C). NH 3 is located in a range of < 10 km; NHs 4, 5 and 11 in a range of 11–25 km; NHs 2, 6, 8, 10, 12 and 13 in a range of 26–50 km and NHs 1, 7 and 9 in a range of 51–75 km from the tertiary care institutions.

The first region enclosed 84 ESBL-*E. coli* isolates from hospital 1 and four NHs (Fig. 1A). Five clonal clusters could be visualized, of which two are large ones with over 10 ST131 ESBL-*E. coli* isolates. The three smaller clusters comprise two isolates each and are within hospital 1 (two clusters of ST2797 and ST38) and NH 7 (a cluster of ST405).

In the second region, the isolates obtained from the hospitalized patients predominate in five of the seven clusters (Fig. 1B). Four clonal clusters (clusters of ST131, ST69, ST23 and ST10) contain isolates obtained from hospital 2 or NH13. The large cluster of ST131 comprises isolates of hospital 2, NH4, NH6 and NH13.

In the third region around hospital 3, a high level of clonality and clustering among the collected ESBL-*E.coli* could be observed (Fig. 1C). Five of nine clonal clusters consist of isolates obtained within hospital 3 (cluster of ST393 and ST131), NH8 (cluster of ST131) and NH12 (cluster of ST127 and ST131). NH3 (seven isolates in total in the two clusters) and hospital 3 (ten isolates in total in the two clusters) have many related isolates and are located in a range of < 10 km. In all regions, there were clusters of isolates in the hospital and the attached nursing homes indicative of transmission between healthcare institutes.

**Fig. 2:**
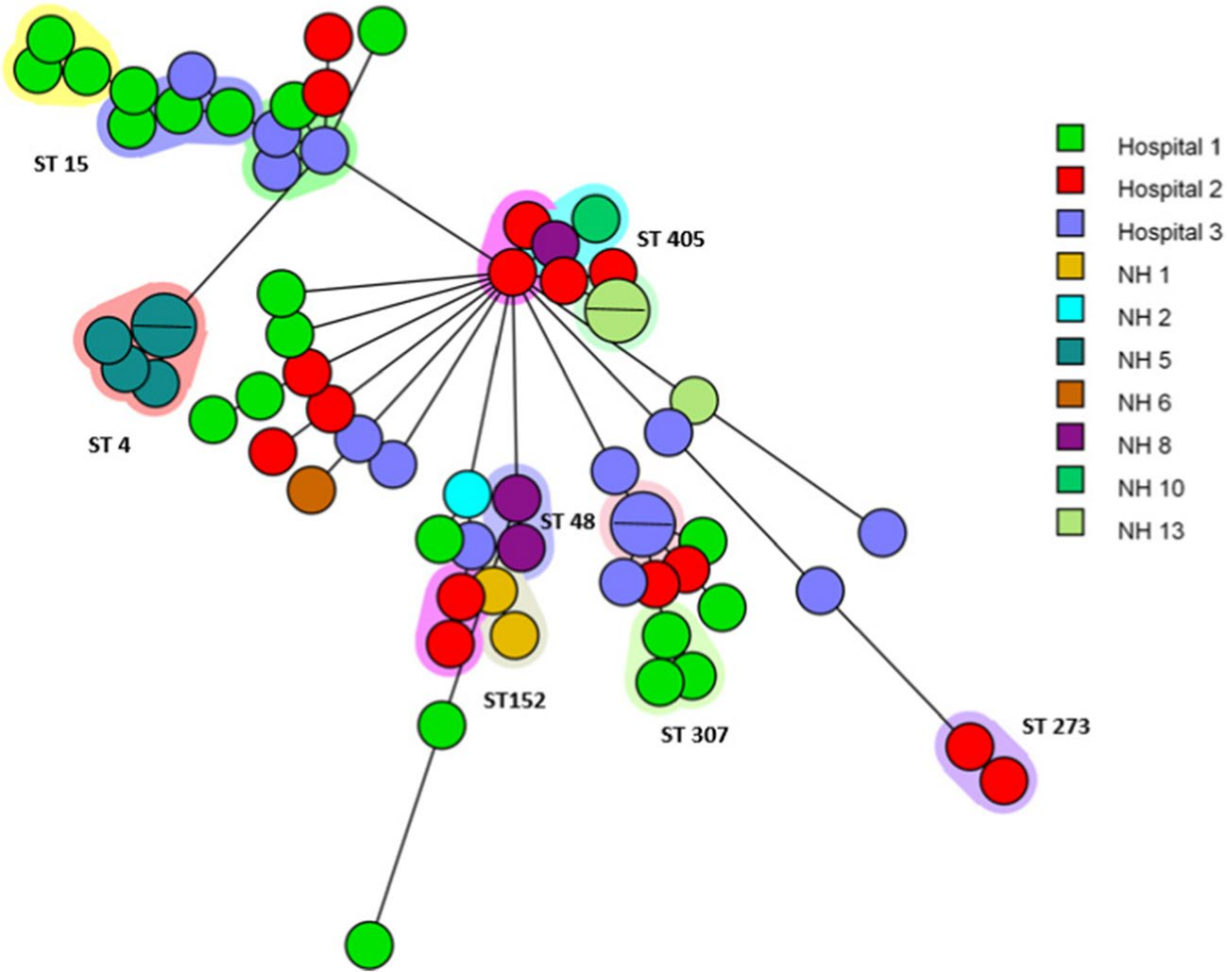
A minimum spanning tree based on wgMLST analysis of 66 ESBL-producing *Klebsiella pneumoniae* isolates. Isolates are represented by circles connected by branches proportional to the allelic distance, colours of the circle represent the location, the shading represent clonal clusters, branches and numbers represent allelic differences between isolates and ST number represent sequence types

A minimum spanning tree of all ESBL-*K. pneumoniae* isolates (*n* = 66) is visualized in Fig. 2. One cluster contains two isolates (ST405) from NH 8 (one) and NH 10 (one) which differ by 3 alleles and have a similarity of 99.9%. NH 8 and 10 are from the same region (within 11–25 km).

### Carbapenemase-producing Enterobacterales

**Fig. 3:**
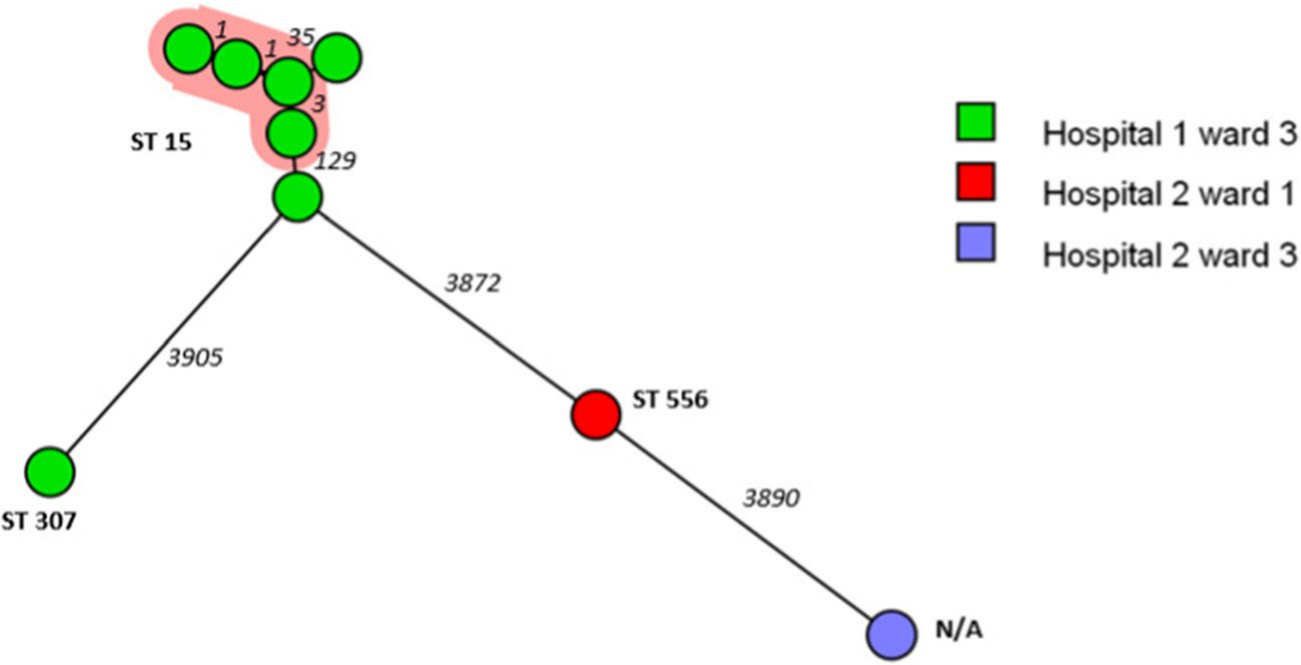
A minimum spanning tree based on wgMLST analysis of 9 OXA-48-positive *Klebsiella pneumoniae* in two hospitals. Isolates are represented by circles connected by branches proportional to the allelic distance, colours of the circle represent the origin location, the shading represent clonal clusters, branches and numbers represent allelic differences between isolates and ST numbers represent sequence types

Twenty-three (1.3%) CPE isolates were detected; all of them originated from hospitalized patients. These CPE isolates consisted of six different species of which *K. pneumoniae* (*n* = 9) and *Citrobacter* spp. (*n* = 7) were predominant. The presence of the *bla*_OXA-48_ gene explained the carbapenem resistance in all but one isolate, a *Citrobacter freundii* strain carrying a *bla*_VIM-4_ resistance gene. All the *bla*_OXA-48_ positive isolates contained the ca. 64kb IncL/M plasmid. Analysis of clonal relatedness of these CPE revealed a cluster of 4 ST15 *K. pneumoniae* CPE in ward 3 of hospital 1 (Fig. 3). In total, 10 *bla*_OXA-48_ producing bacteria of three different species, *K. pneumoniae, Citrobacter* spp. and *E. cloacae* complex were detected in this specific ward of hospital 1.

### Vancomycin-resistant Enterococci

**Fig. 4:**
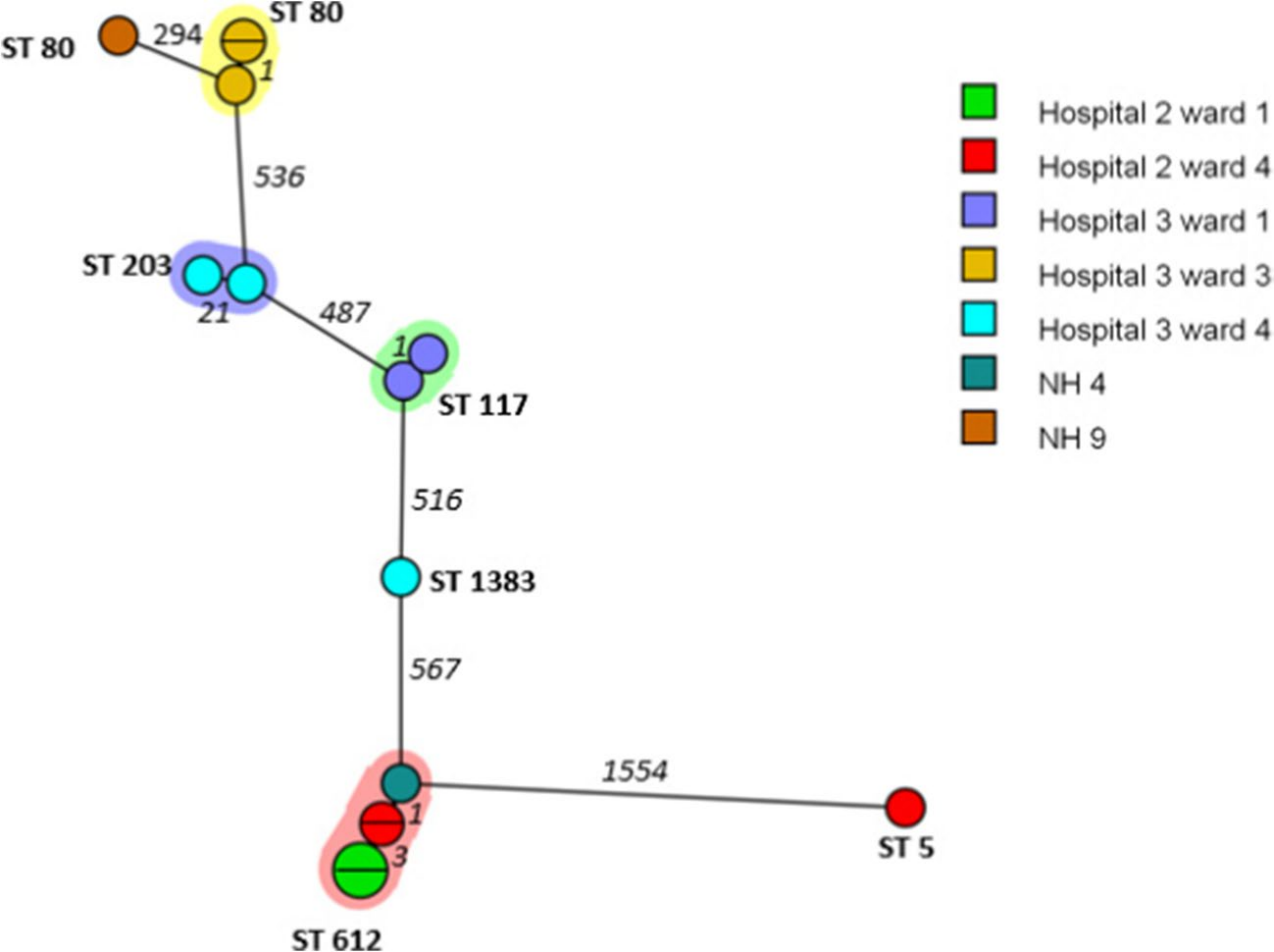
A minimum spanning tree based on wgMLST analysis of 15 vancomycin resistant *Enterococcus faecium* isolates. Isolates are represented by circles connected by branches proportional to the allelic distance, colours of the circles represent the origin location, the shading represent clonal clusters, branches and numbers represent allelic differences between isolates and ST number represent sequence types

Eighteen (0.7%) VRE isolates were detected in the hospitals and NHs. In total, 14 isolates were vanA-positive and four isolates contained vanB. Hospital 3 had three individual clusters of two isolates (ST80, ST203, ST117) on three different wards (Fig. 4). The cluster of five isolates with ST612 consisted of strains of hospital 2 from two different wards and NH 4. Hospital 2 and NH 4 are located within a range of 11–25 km.

## Discussion

The prevalence of ESBL-E was found to be 11.4% in participating hospitals and 14.0% in NHs. Notably, the NH data indicates a rising trend in ESBL-E carriage, with rates increasing from 6.2% in 2011 to 11.3% in 2015 [4]. This upward trajectory may also be observed in Belgian hospitals, as evidenced by a noteworthy increase in the occurrence of ESBL-*E. coli* isolates in clinical samples, escalating from 3.9% in 2005 to 10.1% in 2018 [5].

Among the detected ESBL genes, *bla*_CTX-M-15_ was the most prevalent, followed by other *bla*_CTX-M_-type genes. Rodriguez-Villalobos *et al.* reported an increased presence of *bla*_CTX-M_-type genes in clinical isolates in Belgium in 2008 (54%) compared to 2006 (23%) with 51.5% of the ESBL-*E. coli* isolates and 31% of the ESBL-*K. pneumoniae* isolates harbouring the *bla*_CTX-M-15_ gene in 2008 [6]. In this study, the percentage of *E. coli* containing the *bla*_CTX-M-15_ gene was also 51.5%. However, a remarkable increase could be noted in the *K. pneumoniae* isolates as *bla*_CTX-M-15_ was now present in 90.9% of ESBL-*K. pneumoniae* strains. The predominant ST type among the *bla*_CTX-M-15_ gene harbouring *E. coli* strains is ST131. This pandemic clone is pathogenic with an ability to cause frequent infections in the community, especially in the elderly, and with the remarkable feature of a low level of core genome recombinations [7], as also visible in the minimum spanning tree.

Unlike ESBL-E, CPE’s were not detected in the studied NHs, which is in line with previous Belgian studies conducted in NHs in 2011 [8] and 2015 [4]. In contrast to NHs, CPE could be detected in the hospitals with a mean prevalence of 1% and predominance of *K. pneumoniae* harbouring *bla*_OXA-48_ in line with previous findings [9]. The *bla*_OXA-48_ gene containing CPE isolates in our study (*E. coli, K. pneumoniae, E. cloacae* complex*, Citrobacter* spp., *K. aerogenes* and *M. morgannii*) all harboured the ca. 64kb IncL/M plasmid. The *bla*_OXA-48_ gene on the 64kb IncL/M plasmid is known to be widely distributed in Europe, especially in the Mediterranean area [10]. Therefore, focusing on the clonal spread of one bacterial species may be insufficient to control a plasmid-mediated outbreak [10], as illustrated by the different CPE species (*K. pneumoniae*, *Citrobacter* spp. and *E. cloacae* complex*)* isolated in ward 3 of hospital 1.

In total, 18 VRE isolates were detected with MIC values ranging from 12 to 256 mg/l. The detection of only two VRE in two different NHs is in accordance with the low prevalence described before [4], [11].

To conclude, our study shows, compared to previous studies, that the prevalence of MDRO in Flemish hospitals and NHs has increased over recent years and transmission of ESBL-E, CPE and/or VRE within hospital wards and in and between institutions could be observed. These results re-emphasize the importance of developing an inter-institutional collaboration for infection prevention in the healthcare network.

### Supplementary information

ESM 1

## Data Availability

The data that support the findings of this study are available from the corresponding author, Stefanie van Kleef- van Koeveringe, upon reasonable request.
