# Supplementary material for: Epidemiology and molecular typing of multidrug-resistant bacteria in tertiary hospitals and nursing homes in Flanders, Belgium
Source: Eur J Clin Microbiol Infect Dis. 2023 Nov 16;43(1):187–94. doi: 10.1007/s10096-023-04699-2 (PMC10774642; doi:10.1007/s10096-023-04699-2)
Supplement: Supplementary file 1 — ESM 1 [file 10096_2023_4699_MOESM1_ESM.docx]

Table S1. Thresholds used to determine clonal relatedness based on the similarity of the allelic profiles of two strains.

|  | Similarity (%) threshold | |
| --- | --- | --- |
| Organism | No relatedness | Relatedness |
| *Citrobacter spp* | <90.00 | >98.00 |
| *Enterobacter cloacae* | <98.40 | >99.43 |
| *Enterococcus faecalis* | <98.00 | >99.82 |
| *Enterococcus faecium* | <92.40 | >93.70 |
| *Escherichia coli* | <90.12 | >97.54 |
| *Klebsiella aerogenes* | <95.10 | >97.70 |
| *Klebsiella oxytoca* | <90.00 | >99.13 |
| *Klebsiella pneumoniae* | <96.12 | >99.64 |
